# Prevalence and Associated Factors of Polypharmacy in Nursing Home Residents: A Cross-Sectional Study

**DOI:** 10.3390/ijerph18042037

**Published:** 2021-02-19

**Authors:** Raquel Cadenas, María José Diez, Nélida Fernández, Juan José García, Ana M. Sahagún, Matilde Sierra, Cristina López, Julen Susperregui, Raquel Díez

**Affiliations:** 1 Pharmacology, Department of Biomedical Sciences, Faculty of Health Sciences, Institute of Biomedicine (IBIOMED), University of León, 24071 León, Spain; rcads@unileon.es (R.C.); mjdiel@unileon.es (M.J.D.); mnferm@unileon.es (N.F.); jjgarv@unileon.es (J.J.G.); msiev@unileon.es (M.S.); clopcd@unileon.es (C.L.); rdielz@unileon.es (R.D.); 2 Applied Mathematics, Department of Mathematics, University of León, 24071 León, Spain; jsusl@unileon.es

**Keywords:** aging, elderly, medication, nursing home, polypharmacy, public health

## Abstract

In Spain, there has been a progressive increase in aging. Specifically, Leon has been one of the Spanish provinces with the highest aging index. Polypharmacy is highly prevalent among the elderly, with significant consequences for safety. The aim of this study was to assess the consumption of drugs in a nursing home in the province of Leon and establish the relationship between polypharmacy and the most common comorbid diseases. A descriptive, observational, and cross-sectional study design was used. Residents’ information and treatments were collected by reviewing on medical charts, completed with clinical information obtained from the physician of the nursing home. The mean age of residents was 86.8 years, and 63.8% were female. Mean medical consumption amounted to 7.02 prescriptions. Polypharmacy was observed in 54.9% of residents, and excessive polypharmacy in 22.1%. The most commonly used medications (ATC classification) were those acting on the cardiovascular system (84.4%) and the nervous system (82.8%). A high-level of drug consumption was observed in the nursing home. Interventions should focus on those residents hospitalized the last year and with recent contact with a general practitioner. There is a need to develop a comprehensive monitoring system to assess the quality of prescriptions for nursing home residents.

## 1. Introduction

Aging is a global phenomenon that affects especially the most developed countries. In Spain, it is increasing dramatically: people aged 65 and over represented 19.1% of the Spanish population in 2017, with approximately one-third over 80 years (6.2% of the Spanish population). Moreover, it is estimated that in 2050 Spanish population aged 65 and over will exceed 30%, and if only people aged 80 and over are considered, they will account for 12% of the Spanish population [1]. Castile-Leon is the region with the highest proportion of people over 64 years (24.4%) [2]. In this region, Leon is the second province with the highest aging index (2.41) in 2020 (calculated as the proportion between people over the age of 64 and under age 16) [3]. Aging is a dynamic, complex and continuous process characterized by a rising susceptibility to the occurrence of multiple chronic diseases (comorbidity) that often results in the concomitant use of multiple drug therapies (polypharmacy) for treatment and prophylaxis.

The World Health Organization (WHO) defines polypharmacy as “the administration of many drugs at the same time or the administration of an excessive number of drugs” [4]. Although this term is not uniformly defined in the literature, the most commonly used definition considers that five or more drugs are taken daily [5,6,7,8,9,10]. Polypharmacy in the elderly is increasing and has been identified as a medication safety issue. The risk of an adverse event raises exponentially for five medications [11,12]. Surveys estimate that more than 60% of people over 65 are being prescribed three or more medications on a daily basis, and about 39% have more than five prescriptions [13,14]. Polypharmacy increases the risk of adverse drug-related events in older adults as a higher number of drugs comes with a higher risk of drug–drug interactions. Moreover, the aging process is associated with physiological changes that make the elderly more prone to adverse drug reactions [15].

Nursing home (NH) residents are no exception in polypharmacy. Moreover, they are usually frail and present multiple chronic diseases and a high rate of functional and cognitive impairment. Spain had more than 372,000 beds for nursing and residential care facilities in 2016 [2], and about 4.3% of the elderly were NH residents. They were an average age of 83 years old and 65% dependent on others for daily care needs. Castile-Leon had 45,783 places in residential centers, with an occupancy rate of 100% [2].

Several international experts have recognized the need to perform epidemiological and clinical research in NH [16,17]. It is consequently important to monitor polypharmacy in this group of the elderly population and explore those factors that contribute to it. Given the high percentage of the institutionalized elderly population in Leon, they would become a representative sample to evaluate drug consumption and the prevalence of chronic diseases in order to plan health resources and effective interventions. Hence, the aim of this study was to assess the consumption of drugs in a Spanish nursing home of the province of Leon and to establish if polypharmacy is related to the most common comorbid diseases.

## 2. Materials and Methods

A descriptive, observational, and cross-sectional study was carried out in a Spanish NH located in the province of Leon. Information on institutionalized elderly and their treatments were collected from January to June 2019 by one of the nurses of the NH (first author of the paper), who reviewed on medical charts, completing it with clinical information obtained from the physician of the NH. Retrieved data included demographic characteristics of the residents (age, gender, and origin), length of stay, contacts in the past 2 months with a general practitioner (GP), hospitalization in the past 12 months, pathologies, and medications. Only residents aged ≥65 and institutionalized for at least 1 month were considered. Inclusion criteria for recorded medication were as follows: chronic treatments given orally, by inhalation, or eye drops, administrated for at least 1 month prior to data collection. Treatments initiated less than one month before data collection, complementary, over-the-counter (OTC) medications and nutritional supplements were excluded. Data collection was authorized by the NH directorship. Data were recorded from the NH management software on an Excel sheet. To guarantee the confidentiality of data and the identity of residents, no individual identifiers were used. The Strengthening the Reporting of Observational Studies in Epidemiology (STROBE) Statement was used to report data [18].

The Anatomical Therapeutic and Chemical Classification (ATC) [19] until the 5th level was employed to code medicines. Any combination medicine (multiple component products) was considered as a single medicine. Medicines with different brand names and generics having the same ATC code were considered as one medicine. All medications included in this study needed prescription.

The necessary minimum sample size was estimated in 267 residents, assuming a precision of 0.06, an estimated probability of 0.5 and a significance level of 0.5. The NH was chosen as it exceeds the minimum sample size, obtaining better precision.

### 2.1. Statistical Analysis

Descriptive statistics (frequencies, means, standard deviations, and ranges) were used to characterize the study population. According to previous publications, polypharmacy status was categorized into 3 groups: non-polypharmacy (0–4 medicines), polypharmacy (5–9 medicines) and excessive polypharmacy (at least 10 medicines) [20,21,22,23,24,25,26].

The chi-squared test was employed for the comparison of categorical variables. In addition, logistic regression was performed to identify those variables associated with polypharmacy. The odds ratio (OR) was calculated with their respective 95% confidence intervals (95% CI). A *p*-value of <0.05 was used as the significance level. Data analysis was performed with the statistical package SPSS Statistics version 26 (IBM Corporation, Armonk, NY, USA).

### 2.2. Ethical Considerations

The study was approved by the Institutional Review Board of the Nursing Home and the Ethics Committee of the University of Leon (ULE-0382018) and carried out in accordance with the Declaration of Helsinki.

## 3. Results

Three hundred twenty-six residents were included in this study. Their mean age was 86.8 ± 7.5 years (range 67–107 years), being 63.8% women (Table 1). Residents had mostly lived in the NH less than 5 years (52.2%), they had previously lived in their homes (65.9%), had contact in the past 2 months with a GP (65.0%), and did not require hospitalization in the past 12 months (69.9%). The mean number of medicines used per person was 7.02 ± 3.31 (range: 0–17).

Table 1 also summarizes the characteristics of the sample according to the polypharmacy status. Polypharmacy was observed in 54.9% of residents, and excessive polypharmacy in 22.1%. In these groups, resident profile followed a similar pattern to that of the total study population, though in the excessive polypharmacy group, males rose to 44.4%, three-quarters had contact with a GP in the last two months, and more than half had been hospitalized in the last 12 months.

Multiple comorbidities were observed in most residents, with a median of 7.8 clinical problems per resident (range 0–21). 98.8% had two or more diseases diagnosed by the GP. The pathologies diagnosed for the study population and for each group of polypharmacy are summarized in the supplementary file (Supplementary Materials Table S1). High blood pressure (55.5%), cognitive impairment (34.1%) and bone fractures (34.0%) were the most frequent pathologies diagnosed. When both polypharmacy groups were compared, the prevalence of pathologies clearly increased in the excessive polypharmacy group, especially in the case of constipation (18.4% vs. 34.7%), diabetes (29.1% vs. 44.4%), dyslipidemia (27.4% vs. 42.2%), and bone fractures (31.8% vs. 40.3%). The opposite happened in the case of cognitive impairment (38.5% vs. 30.6%).

**Figure 1:**
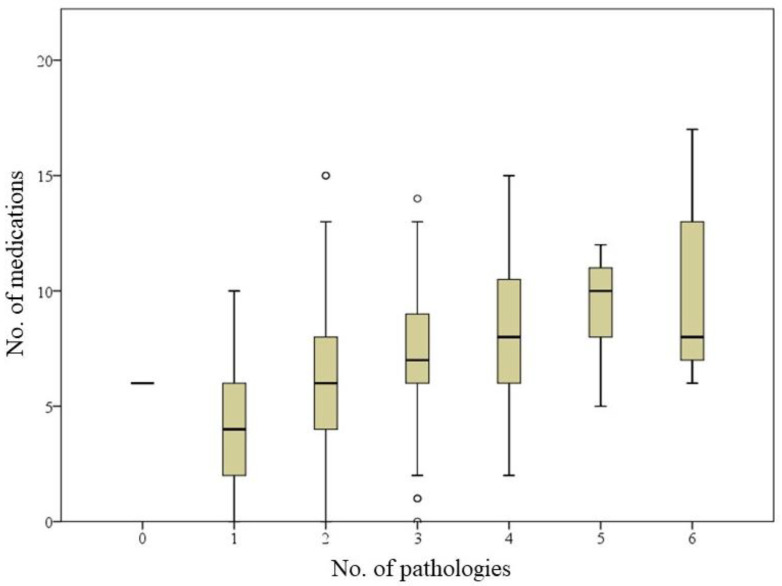
Relationship between polypharmacy and number of pathologies (**0**: no pathology; **1**: 1–3 pathologies; **2**: 4–6 pathologies; **3**: 7–9 pathologies; **4**: 10–12 pathologies; **5**: 13–15 pathologies; **6**: 16 or more pathologies).

Moreover, the number of chronic medications was clearly related to the number of pathologies listed for each resident (Figure 1). Only in the group with the highest number of comorbidities the polypharmacy dropped.

Regarding the pattern of drug consumption by the NH residents with respect to the anatomical group of the ATC classification (first level) (Supplementary Materials Table S2), medicines active on the alimentary tract and metabolism were the most frequently used drugs (85.3%), followed by those acting on the cardiovascular (84.4%) and the nervous (82.8%) systems. A similar profile of consumption was observed when the population was grouped by their level of polypharmacy. Only in the case of group A (alimentary tract and metabolism), its consumption rose to 98.6% in the excessive polypharmacy group.

The ten most commonly used pharmacological subgroups (third level of ATC classification) are shown in Supplementary Materials Table S3. Drugs for peptic ulcer and gastro-esophageal reflux disease were the most commonly used in polypharmacy residents (86.1%) and the second one in the non-polymedicated group (36.0%).

The use of diuretics and antithrombotic agents was also common in the polypharmacy group (57.4% and 56.2%, respectively), with values clearly lower in non-polymedicated residents (18.7% and 16.0%, respectively). Consumption percentages of antidepressants were, however, similar in both groups.

When the 5th level of ATC classification is considered, the most commonly used active ingredients among the elderly in our study included omeprazole (45.7%), furosemide (44.2%), and acetylsalicylic acid (30.4%).

### Multivariate Analysis

Table 2 summarizes the results of the multivariate analysis, identifying those variables associated with polypharmacy. Total polypharmacy (consumption of 5 or more drugs) was directly associated with a higher hospitalization in the last 12 months (OR = 2.0; 95% CI 1.04–3.85). A direct association between excessive polypharmacy and contact with a GP in the last 2 months (OR = 2.23; 95% CI 1.11–4.51) and with hospitalization in the last year (OR = 3.14; 95% CI 1.41–6.97) was also observed. When the three most consumed anatomical groups (ATC first level) were considered (Supplementary Materials Table S4), no significant association was obtained between these groups and the factors assessed, except when the origin of the residents was not known. These residents showed higher consumption of cardiovascular drugs (OR = 3.91; 95% CI 1.02–14.89).

## 4. Discussion

Polypharmacy is a public health problem that is rising in parallel with a longer life expectancy of the population and a higher prevalence of chronic pathologies in older people. The present study examines the prevalence and factors associated with polypharmacy and excessive polypharmacy in a sample of NH residents in the province of Leon (Spain), with one of the most aged populations in this country. Thus, it would reflect a good approach to prescription trends in Spanish NH. Although it has not been possible to associate most of the factors assessed to polypharmacy, the study confirms the high consumption of medications in NH residents, as reported in other studies [24,25,27,28,29,30]. There has been a progressive increase in the number of medications consumed by the older population in recent years, possibly as the result of a combination of several factors, such as increased chronicity and morbidity in this group of the population, but also irrational drug use and increased availability of drugs [31,32,33,34]. On the other hand, NH residents usually have multiple clinical problems and, consequently, the number of medical treatments will be high.

Although the increase in the use of medications may mean greater access to treatments, better approaches to disease and improved quality of life, polypharmacy are becoming a critical problem for national health systems, as it causes a multitude of problems in older people, such as a higher risk of drug–drug interactions and adverse drug reactions, hospitalizations, and mortality if potentially inappropriate medicines are administered [35,36,37,38]. Polypharmacy also increases the risk of vulnerability among the elderly, as does the probability of falls, fractures, cognitive impairment, urinary incontinence and delirium [35].

Increasing age is, however, associated with a lower prevalence of excessive polypharmacy, as in other studies [24,30]. This fact probably reflects the lack of data on the benefits of chronic therapies in very old people [24,27,39] and the reduced use of medications in those persons with a limited life expectancy [40].

Cognitive impairment is also associated with a reduced rate of excessive polypharmacy. As reflected the Beers criteria of the American Geriatrics Society [41] and the STOPP/START criteria [42], there is a need to avoid drugs that may affect cognition or induce delirium and behavioral symptoms when treating older adults with coexisting cognitive impairment, which could explain the lower use of drugs in older people.

As explained previously, the most frequently used active ingredients (5th level) were omeprazole, furosemide, and acetylsalicylic acid. These results are in line with those described by other authors [30,43]. When the ATC classification third level was considered, drug consumption is concentrated in several groups in the polymedicated population. The most consumed pharmacological subgroup was A02B (drugs for peptic ulcer) to prevent those adverse reactions caused by other medicines administered to NH residents. Antithrombotic agents (B01A) and diuretics (C03C) are also among those most consumed drugs, which is in agreement with other authors [24,25,43]. It is also interesting the high use of anxiolytics in the polypharmacy group (43.4%). As reported in other studies, their use is more common among institutionalized people [14,44]. However, this high consumption is a matter of concern, as they should not be prescribed on a long-term basis. Thus, efforts should be made to improve medication usage in NH residents.

Several attempts to reduce polypharmacy in clinical settings have been made [45,46,47]. Some countries like Belgium have published guidance on how to prescribe for older people [25]. In Spain, potentially inappropriate prescribing in older people have been reviewed in accordance with the STOPP/START criteria [48]. This would be one of the strategies that could help to optimize drug therapy. These studies should be carried out periodically, as they allow to assess the actual use of medicines and the associated factors, complementing information obtained from drug utilization studies. On the other hand, non-pharmacological approaches should also be considered. The HALT study has shown a successful and sustainable reduction in regular antipsychotic use based on an individualized deprescribing intervention and education/training of healthcare professionals (GPs, pharmacists, and nurses) [49]. Regarding people with dementia, non-pharmacological approaches have been recommended by international guidelines as first-line treatments, considering pharmacological approaches only when the former fail [50,51]. Among these non-pharmacological approaches, music therapy or psychological treatments have been described [52].

Some limitations need to be recognized in the present study. It was carried out in only one NH, and the sample could not be nationally representative, although the number of institutionalized persons was high. On the other hand, it did not include complementary or OTC drugs, which would imply that the level of polypharmacy could be even higher.

Our study also shows several strengths. Up to our knowledge, it is the first study carried out in a Spanish NH from a geographical area with a high prevalence of aged people in which polypharmacy is extensively studied. We have found only one study in which these factors associated with polypharmacy were analyzed but in non-institutionalized older people from Castile-Leon (Spain) [53]. On the other hand, data were directly retrieved from primary sources (neither questionnaires nor retrospective databases) such as medication charts and information obtained from the physician, being consequently more exhaustive.

Our results provide a high estimate for the prevalence rates of polypharmacy and excessive polypharmacy in NH. Up to 26.6 and 52.0% of residents aged 75–84 and 85–94 showed polypharmacy (29.6 and 56.3% on excessive polypharmacy, respectively), which is worrying. The study confirms that polypharmacy and excessive polypharmacy are unfortunately common among NH residents in Europe [54].

Health professionals can have an important role in reducing polypharmacy in NH by implementing alternatives like deprescribing guidelines or multidisciplinary education in geriatric pharmacotherapy. As an interprofessional team, each member plays a role in helping to reduce medication use and monitor the adverse effects.

The use of multiple medicines may be clinically appropriate for some institutionalized elderly, but it is important to identify those who may be at risk of adverse health outcomes as a result of inappropriate polypharmacy. This is critical to facilitate the deprescribing of inappropriate medications and the optimal use of appropriate medications, but clinical practice guidelines should be periodically updated.

## 5. Conclusions

Our study has shown that polypharmacy is a major problem in the NH assessed. Almost a quarter of the participants aged 75–84, and more than a half of those aged 85–94 are exposed to polypharmacy and excessive polypharmacy, which is of concern. A mean of 7.02 ± 3.31 drugs was consumed by residents. Interventions should be focused on NH residents hospitalized in the last year and those in contact with the GP in the past two months. Attempts to reduce the use of inappropriate drugs should be made. Health professionals play an important role as a checkpoint to evaluate the necessity of drug treatment, especially among the elderly.

## Figures and Tables

**Table 1:** Characteristics of the residents in the nursing home (NH) studied, stratified by the level of polypharmacy.

Characteristic	All (*n* = 326)		Non-Polypharmacy (0–4 Drugs) (*n* = 75)		Polypharmacy (5–9 Drugs) (*n* = 179)		Excessive Polypharmacy (≥10 Drugs) (*n* = 72)	
	%	95% CI	%	95% CI	%	95% CI	%	95% CI
Sex								
Males	36.2	30.9–41.4	38.7	27.7–49.7	31.8	25.0–38.7	44.4	32.9–55.9
Females	63.8	58.9–69.0	61.3	50.3–72.4	68.2	61.3–74.9	55.6	44.1–67.0
Age (years) (*n* = 319)								
65–74	6.3	3.6–8.9	7.0	1.1–13.0	6.2	2.7–9.8	5.6	0.3–11.0
75–84	27.9	23.0–32.8	29.6	18.9–40.2	26.6	20.0–33.1	29.6	19.0–40.2
85–94	52.0	46.6–57.5	47.9	36.3–59.5	52.0	44.6–59.3	56.3	44.9–67.9
≥95	13.8	10.0–17.6	15.5	7.1–23.9	15.2	10.0–20.5	8.5	2.0–14.9
Length of stay (years)								
<5	52.2	46.7–57.6	49.3	38.0–60.7	52.0	44.6–59.3	55.6	44.1–67.0
5–10	29.1	24.2–34.1	34.7	23.9–45.4	26.8	20.3–33.3	29.2	18.7–39.7
10–15	12.3	8.7–15.8	13.3	5.6–21.0	13.4	8.4–18.4	8.3	2.0–14.7
>15	6.4	3.8–9.1	2.7	0–6.3	7.8	3.9–11.8	6.9	1.1–12.8
Origin								
Other NH	17.5	13.3–21.6	13.3	5.6–21.0	16.8	11.3–22.2	23.6	13.8–33.4
Home	65.9	60.8–71.1	66.7	56.0–77.3	68.7	61.9–75.5	58.3	46.9–69.7
Prison	0.6	0–1.46	0	0	0.6	0–1.7	1.4	0–4.1
Unknown	15.9	11.9–19.9	20.0	10.9–29.1	13.9	8.9–19.0	16.7	8.1–22.3
Contact with GP in past 2 months								
No	35.0	29.8–40.2	42.7	31.5–53.9	35.8	28.7–42.8	25.0	15.0–35.0
Yes	65.0	59.9–70.2	57.3	46.1–68.5	64.2	57.2–71.3	75.0	65.0–85.0
Hospitalization in past 12 months (*n* = 249)								
No	61.9	56.6–67.1	73.7	63.7–83.7	62.6	55.5–69.7	47.2	33.7–60.6
Yes	38.1	32.9–43.4	26.3	16.4–36.3	37.4	30.3–44.5	52.8	39.4–66.3

CI: confidence interval; GP: general practitioner.

**Table 2:** Factors associated with polypharmacy among residents in the NH studied (reference category: non-polypharmacy).

Factor	Polypharmacy (5–9 Drugs)	Excessive Polypharmacy (≥10 Drugs)	Total Polypharmacy (≥5 Drugs)
**Odds Ratio (95% CI)**			
Gender Male	1.35 (0.77–2.37)	0.79 (0.41–1.52)	1.15 (0.67–1.95)
Age (years) (*n* = 319)			
75–84	1.19 (0.67–2.11)	1.06 (0.54–2.11)	1.15 (0.66–1.99)
85–94	0.88 (0.50–1.53)	0.75 (0.38–1.46)	0.84 (0.49–1.43)
≥95	1.06 (0.55–2.06)	1.78 (0.74–4.24)	1.21 (0.64–2.30)
Length of stay (years)			
<5	0.73 (0.42–1.25)	0.65 (0.34–1.26)	0.71 (0.42–1.19)
5–10	1.12 (0.64–1.97)	1.05 (0.54–2.06)	1.10 (0.65–1.88)
10–15	0.71 (0.35–1.44)	1.06 (0.43–2.57)	0.79 (0.39–1.57)
Origin			
Home	0.65 (0.32–1.33)	0.81 (0.35–1.88)	0.70 (0.36–1.36)
Unknown	1.12 (0.63–1.99)	0.72 (0.37–1.42)	0.98 (0.57–1.70)
Contact with GP in past 2 months	1.34 (0.77–2.32)	2.23 (1.11–4.51) ^1^	1.53 (0.90–2.60)
Hospitalization in past 12 months (*n* = 249)	1.67 (0.85–3.31)	3.14 (1.41–6.97) ^1^	2.0 (1.04–3.85) ^1^

^1^ significant difference. CI: confidence interval; GP: general practitioner.

## Data Availability

Data are available on request from the corresponding author.

## Supplementary Materials

The following are available online at https://www.mdpi.com/1660-4601/18/4/2037/s1. Table S1: Characteristics of the most frequently diagnosed clinical problems in the NH residents stratified by the level of polypharmacy. Table S2: Characteristics of the anatomical group (1st level ATC) among NH residents stratified by the level of polypharmacy. Table S3: A Characteristics of the 10 most commonly used drug groups (3rd level ATC) among NH residents stratified by the level of polypharmacy. Table S4: Factors associated with the consumption of the three most used anatomical groups in the NH studied (reference category: non-consumption).
