# Supplementary material for: Prevalence and Associated Factors of Polypharmacy in Nursing Home Residents: A Cross-Sectional Study
Source: Int J Environ Res Public Health. 2021 Feb 19;18(4):2037. doi: 10.3390/ijerph18042037 (PMC7922018; doi:10.3390/ijerph18042037)
Supplement: Supplementary file 1 [file ijerph-18-02037-s001.pdf]

Table S1. Characteristics of the most frequently diagnosed clinical problems in the NH residents stratified by the level of polypharmacy.

**Pathology**	**All (*n* = 326)**		**Non-polypharmacy (0–4 Drugs) (*n* = 75)**		**Polypharmacy (5–9 Drugs) (*n* = 179)**		**Excessive Polypharmacy (≥10 Drugs) (*n* = 72)**	
	**%**	**95% CI**	**%**	**95% CI**	**%**	**95% CI**	**%**	**95% CI**
High Blood Pressure	55.5	50.1–60.9	40.0	28.9–51.1	58.7	51.5–65.9	63.9	52.8–75.0
Cognitive impairment	34.1	28.9–39.2	26.7	16.7–36.7	38.5	31.4–45.7	30.6	19.9–41.2
Cataract	31.9	26.8–37.0	25.3	15.5–35.2	33.5	26.6–40.4	34.7	23.7–45.7
Bone fractures	34.0	28.9–39.2	33.3	22.7–44.0	31.8	25.0–38.7	40.3	29.0–51.6
Depression	21.8	17.3–26.3	16.0	7.7–24.3	22.9	16.8–29.1	25.0	15.0–35.0
Constipation	20.2	15.9–24.6	10.7	3.7–17.7	18.4	12.8–24.1	34.7	23.7–45.7
Diabetes	28.8	23.9–33.8	13.3	5.6–21.0	29.1	22.4–35.7	44.4	33.0–55.9
Arthrosis	29.7	24.8–37.7	28.0	17.8–38.2	30.7	24.0–37.5	29.2	18.7–39.7
Urinary incontinence	27.6	22.8–32.5	16.0	77.7–24.3	30.2	23.4–36.9	33.3	22.4–44.2
Dyslipidaemia	28.3	23.4–33.2	17.3	8.8–25.9	27.4	20.8–33.9	42.2	30.8–53.7

CI: confidence interval. Percentages and CI show NH population diagnosed of each disease.

Table S2. Characteristics of the anatomical group (1st level ATC) among NH residents stratified by the level of polypharmacy.

**ATC Group (ATC_1)**	**All (*n* = 326)**		**Non-polypharmacy (0–4 drugs) (*n* = 75)**		**Polypharmacy (5–9 drugs) (*n* = 179)**		**Excessive Polypharmacy (≥10 drugs) (*n* = 72)**	
	**%**	**95% CI**	**%**	**95% CI**	**%**	**95% CI**	**%**	**95% CI**
A Alimentary tract and metabolism	85.3	81.4–89.1	52.0	40.7–63.3	93.8	90.3–97.4	98.6	95.9–100
B Blood and blood forming organs	46.3	40.9–51.7	56.0	44.8–67.2	43.0	35.8–40.3	44.4	33.0–55.9
C Cardiovascular system	84.4	80.4–88.3	82.7	74.1–91.2	84.4	79.0–89.7	86.1	78.1–94.1
G Genito urinary system and sex hormones	8.0	5.0–10.9	9.3	2.8–15.9	6.7	3.0–10.4	9.7	2.9–16.6
H Systemic hormonal preparations, excl. sex hormones and insulins	8.0	5.0–10.9	9.3	2.8–15.9	7.8	3.9–11.8	6.9	1.1–12.8
J Antiinfectives for systemic use	0.9	0–2.0	1.3	0–3.9	0.6	0–1.7	1.4	0–4.1
L Antineoplastic and immunomodulating agents	8.0	5.0–10.9	12.0	4.7–19.4	7.8	3.9–11.8	4.2	0–8.8
M Musculo-skeletal system	18.4	14.2–22.6	18.7	9.9–27.5	19.0	13.3–24.7	16.7	8.1–25.3
N Nervous system	82.8	78.7–86.9	81.3	72.5–90.2	82.7	77.1–88.2	84.7	76.4–93.0
R Respiratory system	16.3	12.3–20.3	12.0	4.7–19.4	18.4	12.8–24.1	15.3	7.0–23.6
S Sensory organs	4.0	1.9–6.1	2.7	0–6.3	3.9	1.1–6.8	5.6	0.3–10.9
V Various	0.3	0–0.9	0	-	0	-	1.4	0–4.1

CI: confidence interval; ATC: Anatomical Therapeutic and Chemical Classification. Percentages and CI show NH population receiving medicines from each ATC group.

Table S3. Characteristics of the 10 most commonly used drug groups (3rd level ATC) among NH residents stratified by the level of polypharmacy.

ATC Code Pharmacological Subgroups (ATC_3)	Non-polypharmacy (0–4 drugs) (*n* = 75)		Polypharmacy (≥5 drugs) (*n* = 251)	
	**%**	**95% CI**	**%**	**95% CI**
A02B Drugs for peptic ulcer and gastro-oesophageal reflux disease	36.0	25.1–46.9	86.1	81.8–90.3
B01A Antithrombotic agents	16.0	7.7–24.3	56.2	50.0–62.3
C03C Diuretics	18.7	9.9–27.5	57.4	51.3–63.5
C09D Angiotensin II antagonists, combinations	17.3	8.8–25.9	18.3	13.5–23.1
C10A Lipid modifying agents, plain	21.3	12.1–30.6	23.9	18.6–29.2
N02B Other analgesic and antipyretics	8.0	1.9–14.1	21.1	16.1–26.2
N05A Antipsychotics	16.0	7.7–24.3	17.9	13.2–22.7
N05B Anxiolytics	28.0	17.8–38.2	43.4	37.3–49.6
N05C Hypnotics and sedatives	16.0	7.7–24.3	14.3	10.1–18.7
N06A Antidepressants	44.0	32.8–55.2	44.2	38.1–50.4

CI: confidence interval; ATC: Anatomical Therapeutic and Chemical Classification. Percentages and CI show NH population receiving medicines from each pharmacological subgroup.

Table S4. Factors associated with the consumption of the three most used anatomical groups in the NH studied (reference category: non-consumption).

Factor	Group A Alimentary tract and Metabolism	Group C Cardiovascular System	Group N Nervous System
	**Odds Ratio (95% CI)**		
Male Gender	0.96 (0.51–1.82)	0.86 (0.46–1.62)	0.73 (0.39–1.36)
	Age (years) (n = 319)		
75–84	1.14 (0.34–3.87)	1.77 (0.50–6.28)	0.65 (0.13–3.14)
85–94	1.64 (0.50–5.35)	1.29 (0.40–4.15)	0.50 (0.11–2.29)
≥95	1.58 (0.39–6.38)	0.97 (0.26–3.63)	0.39 (0.08–1.91)
	Length of stay (years)		
<5	0.32 (0.04–2.50)	1.90 (0.64–5.67)	1.58 (0.49–5.14)
5–10	0.20 (0.03–1.59)	1.43 (0.46–4.45)	0.78 (0.24–2.56)
10–15	0.35 (0.04–3.21)	2.19 (0.55–8.64)	0.94 (0.25–3.58)
	Origin		
Home	0.70 (0.28–1.77)	1.15 (0.55–2.43)	1.28 (0.62–2.66)
Unknown	0.44 (0.15–1.29)	3.91 (1.02–14.89) [1]	1.71 (0.62–4.75)
Contact with GP in past 2 months	1.26 (0.67–2.34)	1.51 (0.82–2.77)	1.14 (0.63–2.07)
Hospitalization in past 12 months (*n* = 249)	2.32 (0.96–5.62)	1.67 (0.81–3.46)	1.34 (0.68–2.65)

[1] significant difference. CI: confidence interval; GP: general practitioner.
